# Severity of Illness and Mortality According to Time of Admission to Intensive Care

**DOI:** 10.1111/aas.70229

**Published:** 2026-03-26

**Authors:** Ville Ihalainen, Anssi Pölkki, Stepani Bendel, Johanna Hästbacka, Sirkku Heino, Matti Reinikainen, the FINNICU Research Group

**Affiliations:** ^1^ Department of Anaesthesiology and Intensive Care Kuopio University Hospital, and University of Eastern Finland Kuopio Finland; ^2^ Department of Anaesthesiology and Intensive Care Tampere University Hospital, Wellbeing Services County of Pirkanmaa, and Tampere University, Faculty of Medicine and Health Technology Tampere Finland; ^3^ Department of Anaesthesiology and Intensive Care North Karelia Central Hospital Joensuu Finland

**Keywords:** admission hours, intensive care, morning, mortality, risk

## Abstract

**Introduction:**

Staffing at hospital wards is at its lowest during nighttime, which may endanger prompt recognition of the need for intensive care. High severity of illness of patients admitted to the intensive care unit (ICU) in the morning may reflect delayed referral for intensive care during the night. We investigated whether severity of illness at ICU admission is dependent on admission time.

**Methods:**

We analysed data from 25 Finnish ICUs between 2005 and 2022, utilising the national ICU registry. We excluded readmissions, children, non‐emergency admissions, cardiac surgery patients and patients admitted for the purpose of organ donation. We explored the severity of illness, as quantified with the Simplified Acute Physiology Score (SAPS) II score, and in‐hospital mortality according to admission hours. We also conducted linear and logistic regression analyses adjusting for age, sex, admission type, source of admission, diagnosis category and severity of illness.

**Results:**

The study population comprised 131,175 ICU patients. The mean (± SD) SAPS II score at admission was 37.9 ± 17.4, and overall in‐hospital mortality was 14.7%. The mean SAPS II score was 39.0 ± 17.9 for morning admissions (6–12 a.m.) and 37.6 ± 17.2 during other times (*p* < 0.001). The corresponding in‐hospital mortalities were 17.5% and 13.9%, respectively (*p* < 0.001). After adjusting for differences in patient characteristics, morning admissions remained independently associated with higher SAPS II score (mean difference 0.87 points, 95% CI, 0.65–1.09) and in‐hospital mortality (OR 1.17, 95% CI, 1.13–1.21).

**Conclusions:**

Patients admitted to intensive care during morning hours experienced higher severity of illness and higher in‐hospital mortality.

**Editorial Comment:**

This analysis, from the national ICU database in Finland, shows that cases with morning admission to the ICU appear to have more severe illness compared to those admitted at other times of day. The authors consider if there might be some nighttime delay for recognition of ICU need which could contribute to this observation.

## 1 Introduction

In a high‐quality healthcare system, the need for intensive care due to critical illness should be promptly recognised at any hour, and the quality of intensive care should remain consistent regardless of when treatment is initiated.

Admission to general hospital wards during the night is associated with higher risk of death [1]. Moreover, nighttime work is associated with less frequent vital sign monitoring and reduced use of Early Warning Score (EWS) on hospital wards [2, 3].

Although nighttime admissions to the ICU have not been associated with risk of death [4], data examining possible delays in recognition of critical illness and subsequent changes in severity of illness and mortality are scarce. Our hypothesis was that recognition of the need for intensive care may be delayed during nighttime hours, which would result in patients admitted to the ICU in the morning being more severely ill than those admitted at other times.

## 2 Methods

### 2.1 Study Design

We conducted a retrospective observational cohort study analysing the association between admission time, severity of illness and in‐hospital mortality. Utilising the national ICU registry, the Finnish Intensive Care Consortium (FICC) database [5], we analysed data on admissions to ICUs between 2005 and 2022. The FICC registry includes 25 Finnish ICUs of varying sizes. For each ICU admission, data on age, sex, premorbid functional status, severe comorbidities, diagnosis of the acute illness, severity of physiological abnormalities, ICU treatments and outcome are recorded. In Finland, there are five university hospitals, and certain treatments (e.g., cardiac surgery, intracranial neurosurgery, endovascular treatment of intracranial vascular pathologies) are centralised and only provided in university hospitals. FICC classifies non‐university central hospital ICUs into large and small ICUs based on the median size of the ICU and hospital. A large central hospital ICU has at least six beds and a referral population of at least 120,000.

We included all ICU admissions recorded in the national registry. We excluded readmissions (repeated ICU admission of the same person), children, elective (non‐emergency) admissions, cardiac surgery patients and patients admitted for the purpose of organ donation.

We used Simplified Acute Physiology Score (SAPS) II to quantify the severity of illness [6]. In analyses, we used admission time both hourly, and divided into four time windows: morning (6–12 a.m.), afternoon (12–6 p.m.), evening (6 p.m.–12 a.m.) and night (12–6 a.m.) In addition, we grouped admission times into two time windows: morning and the rest of the day. We used diagnostic categories according to the Acute Physiology and Chronic Health Evaluation (APACHE) III system [7].

The primary outcome of the study was severity of illness according to admission hour, and the secondary outcome was in‐hospital mortality according to admission hour. Sensitivity analyses included severity of illness and mortality according to admission hour in subgroups based on diagnostic group, hospital size, year of ICU admission and source of ICU admission. In‐hospital mortality was based on vital status at the time of discharge from the hospital where the intensive care had been provided.

The Finnish Social and Health Data Permit Authority Findata approved the use of registry data for this study (Dnro/THL/1743/14.06.00/2024). The requirement for patient consent was waived in accordance with Finnish legislation due to the non‐interventional observational nature of the study.

We report the study according to the STrengthening the Reporting of OBservational studies in Epidemiology (STROBE) guidelines [8].

### 2.2 Statistical Methods

We report frequencies (*n*), percentages (%), mean or median values and standard deviation or interquartile ranges. For group differences between admission times, we report *p* values based on analysis of variance (ANOVA), *t*‐test, or chi‐squared test, as appropriate. We used Bonferroni correction for multiple comparisons. To describe yearly trends, we report mean difference in SAPS II score and mortality with 95% confidence intervals. We used linear and logistic regression to assess the association between admission time and the SAPS II score and in‐hospital mortality, respectively. In the regression analyses, we used admission time categorised into two categories (morning admissions and admissions during other times). The regression model for severity of illness (SAPS II score) at admission included admission time, source of admission (type of department where the patient came from) and diagnosis category as covariates. All predictors in the linear model were categorical and therefore the linearity assumption was not applicable. In graphical assessment of normality of residuals and homoscedasticity, no violation of model assumptions was observed. The model for in‐hospital mortality included the same covariates, and age and admission type (post‐operative or medical). To explore the independent, that is, severity of illness‐adjusted association between admission time and mortality, we used the same multivariable logistic regression model as described above, adding the SAPS II score as a covariate but excluding age and admission type that are components of the SAPS II score. In logistic models, the linearity of continuous variables was assessed graphically on the logit scale, and no violations of log‐linearity were observed. Overall fit of the models was assessed using area under the receiver operating characteristic curve (AUROC) and calibration curves (Figures S1–S4). The models showed excellent internal calibration with moderate to high accuracy. For regression analyses, complete case data were used, since the amount of missing data was very low (Table S1). All *p* values were two‐sided, and *p* values smaller than 0.05 were considered statistically significant. For the statistical analyses, we used R, version 4.4.3 (R Core Team, Vienna, Austria).

## 3 Results

### 3.1 Study Population

**FIGURE 1:**
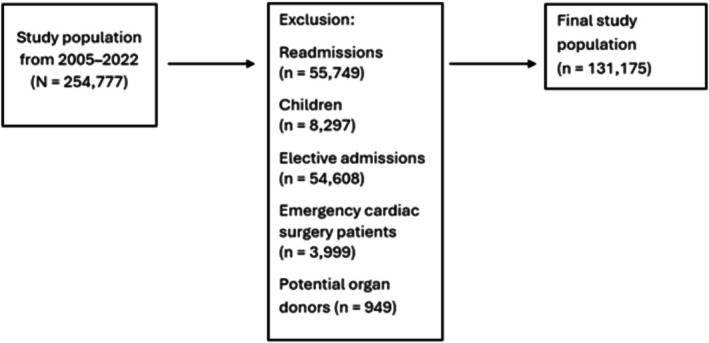
Study flowchart.

The initial study population comprised 254,777 admissions to 25 Finnish ICUs between 2005 and 2022. There were no missing data for admission time. Figure 1 presents the reasons for exclusions. The final study population consisted of 131,175 patients.

Background characteristics of the study population and the distribution to diagnostic categories across admission times are presented in Tables 1 and 2.

**TABLE 1:** Background characteristics of the study population.

	Morning admissions, *n* (%)	Afternoon admissions, *n* (%)	Evening admissions, *n* (%)	Night admissions, *n* (%)
Number of admissions	28,467 (21.8)	37,706 (28.9)	40,032 (30.6)	24,484 (18.7)
Age, years	63 (49–73)	64 (51–74)	63 (49–73)	60 (44–72)
Sex, male	17,820 (62.6)	23,151 (61.4)	24,499 (61.2)	15,351 (62.7)
ICU category*				
Small non‐university	2863 (10.1)	3982 (10.6)	4219 (10.5)	2209 (9.0)
Large non‐university	6984 (24.5)	8475 (22.5)	9123 (22.8)	5513 (22.5)
University	17,368 (61.0)	23,443 (62.2)	24,816 (62.0)	15,505 (63.3)
Admission type*				
Post‐operative	6024 (21.1)	9754 (25.9)	10,857 (27.1)	6149 (25.1)
Medical	22,438 (78.9)	27,945 (74.1)	29,172 (72.9)	18,330 (74.9)
Source of admission*				
Hospital ward	6969 (24.5)	6466 (17.1)	5497 (13.7)	3495 (14.3)
Emergency department	12,084 (42.5)	16,919 (44.9)	20,720 (51.8)	13,331 (54.5)
Operating theatre	5673 (19.9)	9327 (24.7)	10,171 (25.4)	5691 (23.2)
Intermediate care unit	1821 (6.4)	1816 (4.8)	1446 (3.6)	9,12 (3.7)
ICU transfer	325 (1.1)	579 (1.5)	269 (0.7)	121 (0.5)

*Note:* Data presented as absolute numbers and percentages for each admission time window or as medians with interquartile ranges (IQR). *p < 0.001 (chi‐square test).

Abbreviation: ICU, intensive care unit.

**TABLE 2:** Distribution of diagnostic categories across admission times.

	Morning admissions, *n* (%)	Afternoon admissions, *n* (%)	Evening admissions, *n* (%)	Night admissions, *n* (%)
Number of admissions	28,467	37,706	40,032	24,484
Post‐operative	6024 (21.1)	9754 (25.9)	10,857 (27.1)	6149 (25.1)
Gastrointestinal	2049 (7.2)	3179 (8.4)	3358 (8.4)	1859 (7.6)
Neurologic	1104 (3.9)	1868 (4.9)	2272 (5.7)	1385 (5.6)
Other operative	531 (1.9)	915 (2.4)	799 (2.0)	441 (1.8)
Respiratory	356 (1.2)	711 (1.9)	766 (1.9)	281 (1.1)
Trauma	655 (2.3)	922 (2.4)	1354 (3.4)	900 (3.7)
Vascular	1350 (4.7)	2155 (5.7)	2294 (5.7)	1268 (5.2)
Medical	22,438 (78.9)	27,945 (74.1)	29,172 (72.9)	18,330 (74.9)
Cardiovascular/vascular	4639 (16.2)	5550 (14.7)	5148 (12.8)	3067 (12.5)
Gastrointestinal	2129 (7.5)	2486 (6.6)	2371 (5.9)	1567 (6.4)
Intoxication	1239 (4.3)	1257 (3.3)	1890 (4.7)	1802 (7.3)
Metabolic/renal/hematologic	1729 (6.1)	2654 (7.0)	2889 (7.2)	1607 (6.5)
Neurologic	3181 (11.1)	4653 (12.3)	5042 (12.5)	2825 (11.5)
Other non‐operative	1238 (4.3)	1431 (3.8)	1624 (4.0)	1071 (4.4)
Respiratory	4568 (16.0)	4945 (13.1)	4694 (11.7)	2927 (11.9)
Sepsis	2028 (7.1)	2405 (6.3)	2280 (5.7)	1267 (5.2)
Trauma	1671 (5.8)	2575 (6.8)	3251 (8.1)	2217 (9.0)

*Note:* Data presented as absolute numbers (percentages) for each admission time window. Classification based on Acute Physiology and Chronic Health Evaluation (APACHE) III diagnostic categories. *p* < 0.001 (chi‐square test).

### 3.2 Severity of Illness and Mortality

**FIGURE 2:**
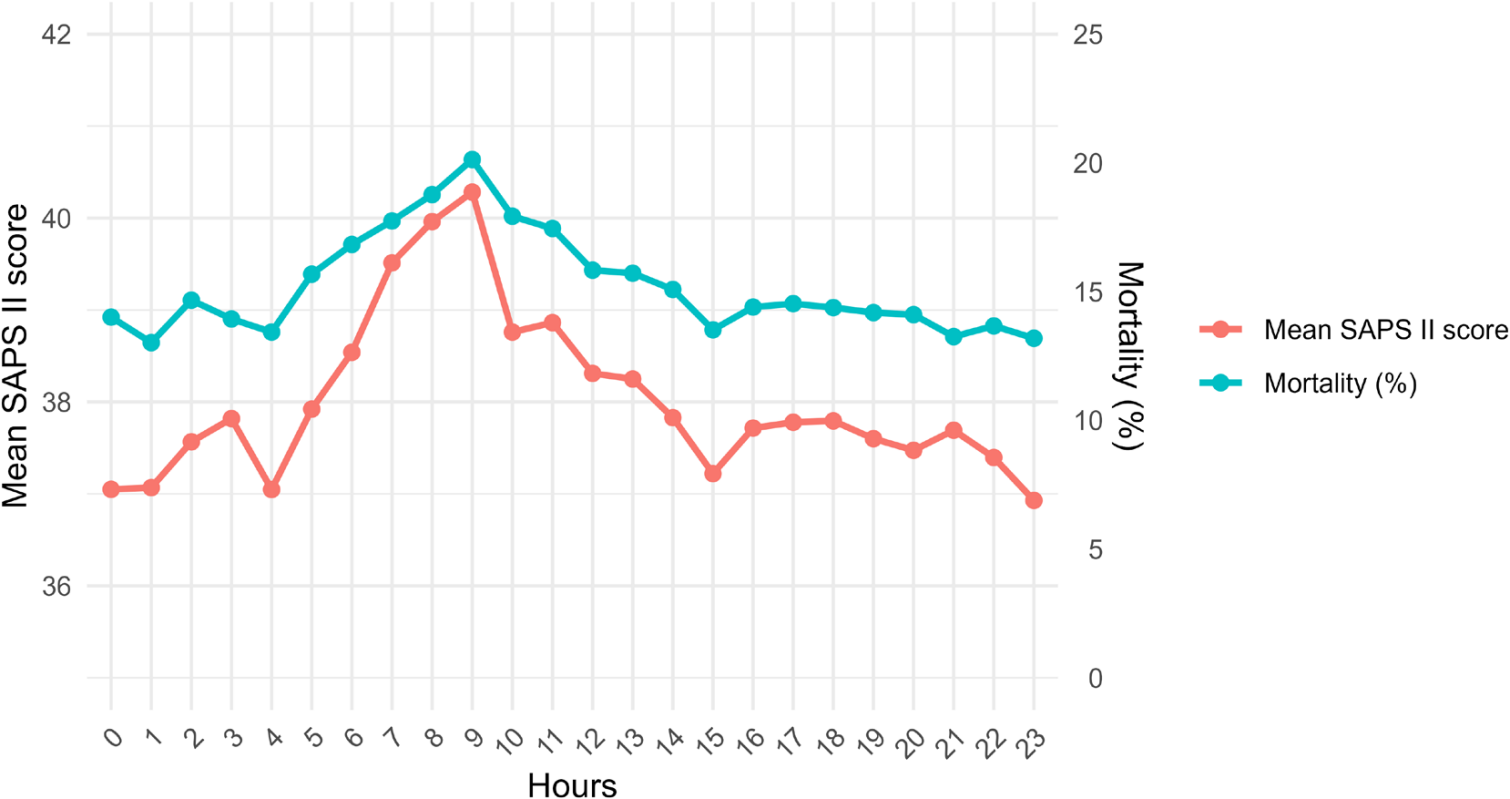
Simplified Acute Physiology Score (SAPS) II and in‐hospital mortality according to admission hours.

The overall mean (± SD) SAPS II score at admission was 37.9 ± 17.5 and in‐hospital mortality was 14.7%. Both SAPS II score and mortality started to increase from early morning and continued to rise until the admission time 9 a.m. (Figure 2).

The mean SAPS II score was 39.0 ± 18.0 for morning admissions, 37.8 ± 17.1 for afternoon admissions, 37.5 ± 17.4 for evening admissions and 37.4 ± 17.7 for night admissions, *p* < 0.001. Corresponding in‐hospital mortalities were 17.6%, 16.7%, 13.8% and 14.0%, respectively, *p* < 0.001. When each admission time window was compared against the rest of the day, only morning admissions were associated with significantly higher SAPS II scores (39.0 ± 18.0 vs. 37.6 ± 17.3, *p* < 0.001). Also, only morning admissions, as compared with admissions during other time windows, were associated with higher in‐hospital mortality (17.6% vs. 14.2%, *p* < 0.001).

Morning admissions were associated with higher SAPS II scores and higher in‐hospital mortality regardless of hospital size (Table 3).

**TABLE 3:** SAPS II scores and in‐hospital mortality between morning (6–12 a.m.) admissions and admissions during other times, categorised by admission type and ICU category.

	SAPS II score, morning admissions	SAPS II score, admissions during other times	Adjusted *p* value	Mortality, morning admissions (%)	Mortality, admissions during other times (%)	Adjusted *p* value
All	39.0 ± 18.0	37.6 ± 17.4	< 0.001	17.6%	14.2%	< 0.001
Admission type						
Post‐operative admissions	37.3 ± 15.5	37.2 ± 15.2	0.60	12.1%	10.7%	0.16
Medical admissions	39.5 ± 18.6	37.7 ± 18.1	< 0.001	19.0%	15.2%	< 0.001
ICU category						
Small non‐university hospital ICU	37.9 ± 17.6	36.1 ± 16.5	< 0.001	18.5%	13.3%	< 0.001
Large non‐university hospital ICU	41.3 ± 18.4	39.8 ± 18.1	< 0.001	19.2%	16.0%	< 0.001
University hospital ICU	39.1 ± 17.9	37.8 ± 17.2	< 0.001	17.7%	14.3%	< 0.001

*Note:* Data presented as absolute numbers, means with standard deviations ± SD and percentages. Bonferroni correction was used to adjust for multiple comparisons.

Abbreviations: ICU, intensive care unit; SAPS II, Simplified Acute Physiology Score.

Morning admissions were associated with higher illness severity and higher mortality in medical (non‐operative) patients. In contrast, there were no statistically significant differences between morning admissions and admissions during other times in post‐operative patients (Table 3).

A decreasing trend in both the mean SAPS II score and mortality was observed over the study period (Figure S5). When the analyses were stratified according to calendar years, the difference between morning admissions and admissions during other times in SAPS II score was statistically significant for most years. However, the difference decreased towards the end of the study period (Figure S6). Likewise, the difference between morning admissions and admissions during other times in crude mortality was statistically significant for most years (Figure S7).

### 3.3 Severity of Illness and Mortality After Adjustment for Patient Characteristics

Patients admitted at different times of day differed in many respects (Tables 1 and 2). Notably, the proportion of patients admitted for non‐operative cardiovascular or respiratory problems was higher in the morning than during other times. However, even after adjustments for differences in patient characteristics, morning admission remained an independent predictor of higher SAPS II scores (mean difference between morning admissions and other times, 0.87 points, 95% confidence interval (CI) 0.65–1.09) and higher in‐hospital mortality (adjusted odds ratio 1.17, 95% CI, 1.13–1.21).

Higher severity of illness and higher mortality for morning admissions was observed in patients admitted from hospital wards, emergency departments and following transfers from other ICUs, but not in patients admitted from operating theatres or intermediate care units (Figures S8–S12).

The impact of morning admission on severity of illness and mortality varied across APACHE III diagnostic categories (Table S2). SAPS II scores were higher for morning admissions in all medical (non‐operative) diagnostic categories, except for APACHE III categories ‘intoxication’ and ‘metabolic/renal/hematologic’ and in post‐operative APACHE III categories ‘respiratory’ and ‘vascular’, but not in other post‐operative patients. The difference in mortality between morning admissions and admissions during other times of the day was statistically significant in all medical diagnostic categories except for intoxications, whereas there was no statistically significant difference in post‐operative categories.

When differences in SAPS II scores were adjusted for, morning admissions remained independently associated with higher in‐hospital mortality in the overall patient population (adjusted odds ratio 1.10, 95% CI, 1.06–1.15). However, when diagnostic categories were studied separately, severity of illness‐adjusted mortality was statistically significantly higher for morning admissions only in patients with a medical (non‐operative) diagnosis of respiratory disease, sepsis and trauma (Table S3).

## 4 Discussion

We conducted this registry‐based study to assess the associations between admission time, severity of illness and in‐hospital mortality. Patients admitted during morning hours (6–12 a.m.) were more severely ill, and they experienced higher in‐hospital mortality as compared to patients admitted during other times. This pattern was consistent across hospitals of different sizes. However, the difference in SAPS II scores between patients admitted in the morning and those admitted during other times decreased towards the end of the study period.

The absolute differences in SAPS II scores were fairly small between patients admitted at different times. However, in the mid‐range of disease severity in our study population, the risk of death increases steeply even with small increases in the score, and therefore the difference is clinically meaningful. This aligns with our finding that there was a considerable difference in mortality between morning admissions and admissions during other times.

We observed higher severity of illness and higher mortality for morning admissions than admissions during other times in patients admitted from hospital wards and emergency departments, but not for patients admitted from operating theatres or intermediate care units.

Several studies have demonstrated an association between delayed admission to the ICU and increased mortality [9, 10, 11, 12, 13], while other studies have failed to find a relationship [14, 15]. However, several of these studies have primarily focused on patients already identified as needing intensive care but whose admission has been delayed due to limited bed availability [9, 10, 11, 14, 15]. Studies focusing on timely onset of appropriate treatment or time from vital sign deterioration to ICU admission have established a clear association between prompt ICU admission and improved survival [12, 13].

EWS systems have been introduced to help recognising critically ill patients [16]. The principle of all EWSs is to measure essential vital signs and assign points based on their deviation from normal. In Finland, the National Early Warning Score (NEWS) was gradually implemented into routine clinical use during the study period, and it has been proven to have an excellent ability to detect critically ill patients [17]. Accordingly, we observed a decreasing trend in the difference in SAPS II scores between morning admissions and admissions during other times.

Delays in recognising the need of intensive care during the night might be a possible explanation for the observed peak in severity of illness among morning admissions. Lower staffing levels in wards at night might be a plausible explanation for this. Previous evidence suggests less frequent vital sign monitoring and EWS utilisation in wards during nights [2, 3]. It is noteworthy that severity of illness at ICU admission was not dependent on admission time in patients admitted from operating theatres or intermediate care units. Staffing levels in these units are typically strong even during nighttime hours. Our results raise concern that the ability to recognise critical illness and promptly initiate intensive care during nighttime may be less than perfect in Finnish hospital wards.

Theoretically, higher mortality of patients admitted to intensive care during morning hours might be a result of arrival of more severely ill patients to emergency departments during nights or mornings. However, two studies evaluating this question did not demonstrate such an association [18, 19].

The difference in risk of death between morning admissions and admissions during other times decreased but remained statistically significant after adjusting for severity of illness in some, but not in most diagnostic categories. The increased mortality risk might be explained by residual confounding, such as availability of medical emergency team or ICU beds, ward and ICU staffing, delays in diagnostics or differences in end‐of‐life decision making between morning and rest of day. Also, while the SAPS II score captures changes in acute physiology, it does not account for many chronic illnesses and previous functional capacity, which are important factors in mortality prediction [20, 21].

## 5 Strengths and Limitations

Strengths of our study include the use of a large, nationwide database comprising data from 25 ICUs. The data were obtained over 18 consecutive years and covered a wide range of ICUs, from those with only a few beds to large university hospital ICUs with specialised intensive care.

We acknowledge that our study has limitations. The retrospective nature of the study makes causal interpretation impossible. Moreover, the exact hospital admission time or NEWS score preceding ICU admission is not recorded in the database. Thus, we were not able to measure time from hospital admission to intensive care or to compare NEWS scores recorded across different admission times. Admission to intensive care is influenced by local policies, and results of this study may not be generalisable outside of Finland.

## 6 Conclusion

Morning‐hour admissions to intensive care were associated with higher illness severity and increased mortality. This phenomenon affected mainly patients who were admitted from hospital wards with medical diagnoses.

## Author Contributions

**Ville Ihalainen:** data curation, formal analysis, writing – original draft. **Anssi Pölkki:** conceptualization, methodology, writing – review and editing. **Stepani Bendel:** resources, writing – review and editing. **Johanna Hästbacka:** funding acquisition, methodology, writing – review and editing. **Sirkku Heino:** conceptualization, writing – review and editing. **Matti Reinikainen:** funding acquisition, conceptualization, methodology, project administration, resources, supervision. All authors read and approved the final manuscript.

## Funding

This study was supported by funding from the University of Eastern Finland and research grants from the Finnish Society of Anaesthesiologists to Ville Ihalainen for PhD studies. The FINNICU study has received the State Research Funding for university‐level health research, Kuopio University Hospital, Wellbeing Service County of North Savo, project number 507T052.

## Conflicts of Interest

The authors declare no conflicts of interest.

## Supporting information


**Data S1:** aas70229‐sup‐0001‐Supinfo.docx.

## Data Availability

Research data are not shared.
