# Supplementary material for: Severity of Illness and Mortality According to Time of Admission to Intensive Care
Source: Acta Anaesthesiol Scand. 2026 Mar 26;70(5):e70229. doi: 10.1111/aas.70229 (PMC13021569; doi:10.1111/aas.70229)
Supplement: Supplementary file 1 — Data S1: aas70229‐sup‐0001‐Supinfo.docx. [file AAS-70-0-s001.docx]

Supplementary file to

**Severity of illness and mortality according to time of admission to intensive care**

by Ville Ihalainen^1^, Anssi Pölkki^1^, Stepani Bendel^1^, Johanna Hästbacka^2^, Sirkku Heino^3^, and Matti Reinikainen^1^; on behalf of the FINNICU research group

Affiliations: ^1^Department of Anaesthesiology and Intensive Care, Kuopio University Hospital, and University of Eastern Finland; ^2^ Department of Anaesthesiology and Intensive Care, Tampere University Hospital, Wellbeing Services County of Pirkanmaa, and Tampere University, Faculty of Medicine and Health Technology, Tampere, Finland; ^3^Department of Anaesthesiology and Intensive Care, North Karelia Central Hospital; all in Finland.

Content:

1. AUROC curves of used logistic regression models,

eFigures 1-2 pages 2-3

1. Calibration curves of used logistic regression models,

eFigures 3-4 page 4

1. SAPS II scores and in-hospital mortality over the study period,

eFigure 5 page 5

1. Difference in SAPS II scores and in-hospital mortality between

morning admissions and admissions during other times,

categorised by admission year, eFigures 6-7 page 6

1. SAPS II scores and in-hospital mortality according to admission

hours, categorised by source of admission, eFigures 8-12 pages 7-11

1. Percentages of missing data, eTable 1 page 12
2. Difference in SAPS II scores and in-hospital mortality between

morning admissions and admissions during other times,

categorised by diagnostic category, eTable 2 page 13

1. Severity of illness adjusted in-hospital mortality difference between

morning admissions and admissions during other times,

categorised by diagnostic category, eTable 3 page 14

1. The FINNICU research group pages 15-16


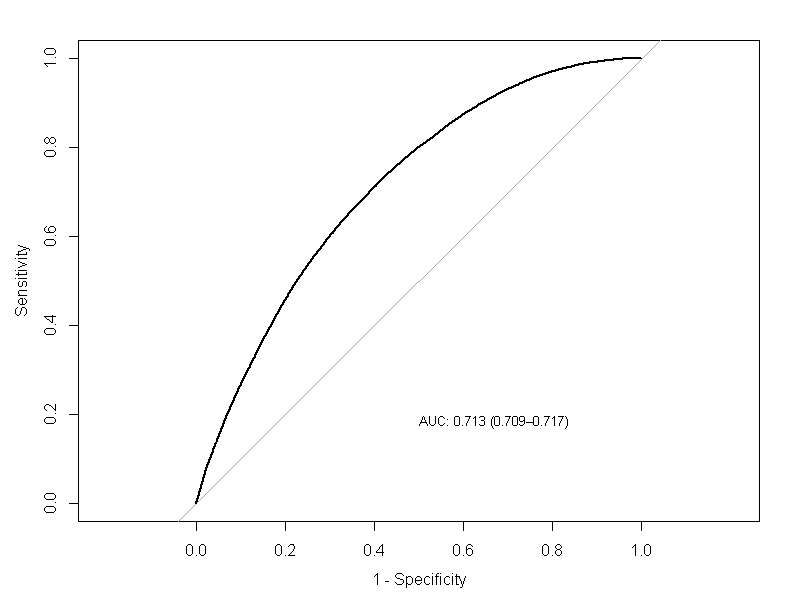


**eFigure 1**. Area under the receiver operating characteristic curve (AUROC) for the logistic regression model with in-hospital mortality as the outcome. Covariates in the model include admission time (morning vs. rest of the day), source of admission (type of department where the patient came from), diagnosis category, age and admission type (post-operative or medical).


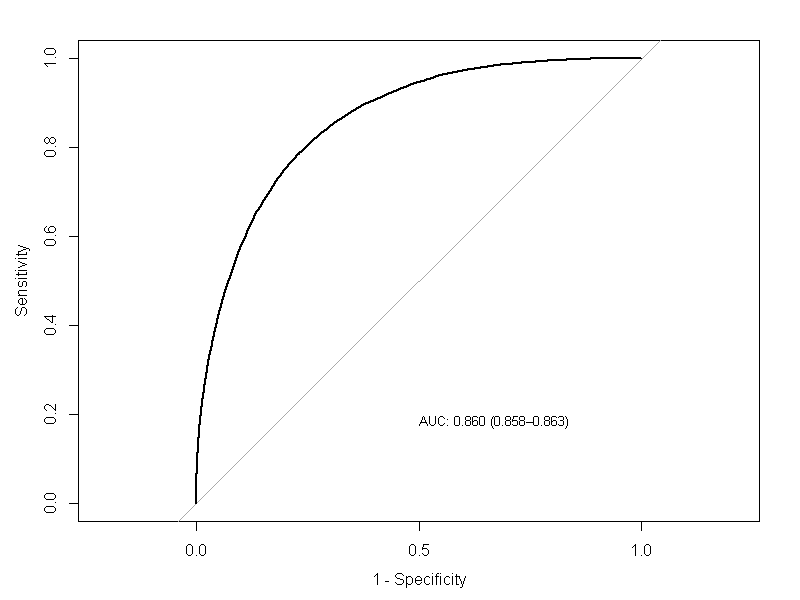


**eFigure 2.** Area under the receiver operating characteristic curve (AUROC) for the logistic regression model with in-hospital mortality as the outcome. Covariates in the model include Simplified Acute Physiology Score (SAPS) II score, admission time (morning vs. rest of the day), source of admission (type of department where the patient came from) and diagnosis category.


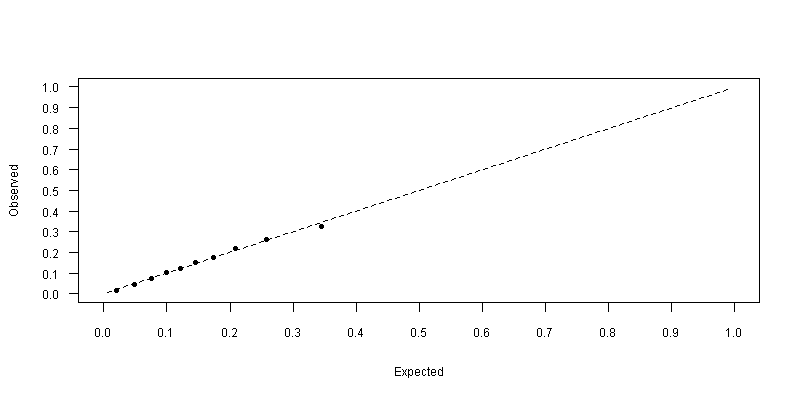


**eFigure 3.** Calibration curve for the logistic regression model with in-hospital mortality as the outcome. Covariates in the model include admission time (morning vs. rest of the day), source of admission (type of department where the patient came from), diagnosis category, age and admission type (post-operative or medical).


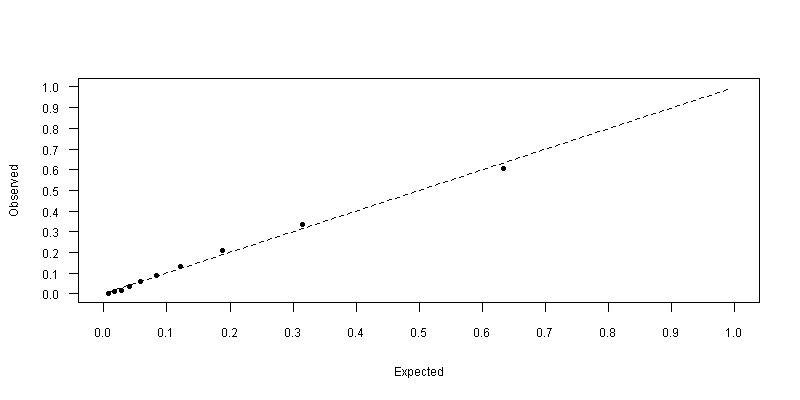


**eFigure 4.** Calibration curve for the logistic regression model with in-hospital mortality as the outcome. Covariates in the model include Simplified Acute Physiology Score (SAPS) II score, admission time (morning vs. rest of the day), source of admission (type of department where the patient came from) and diagnosis category.


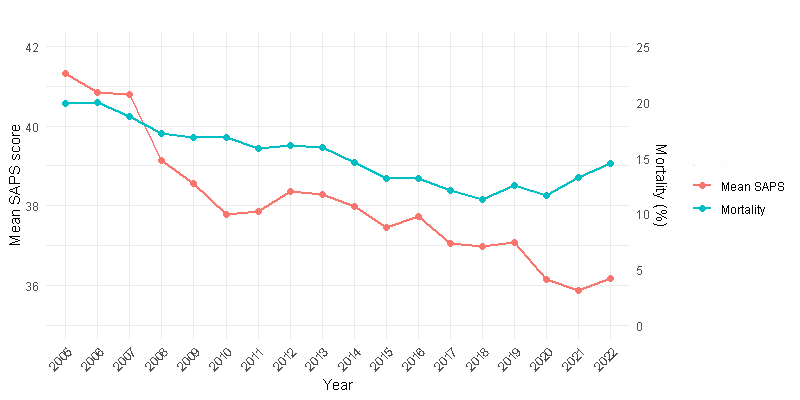


**eFigure 5.** Simplified Acute Physiology Score (SAPS) II and mortality (%) over the study period.


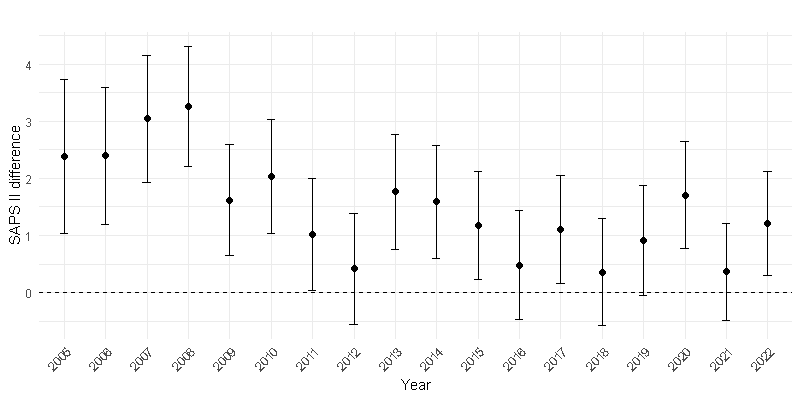


**eFigure 6.** Absolute difference in Simplified Acute Physiology Score (SAPS) II with 95 % confidence intervals between morning admissions and admissions during other times, categorised by admission year. Mean decrease 0.12 points per year (95% CI 0.05 - 0.18).


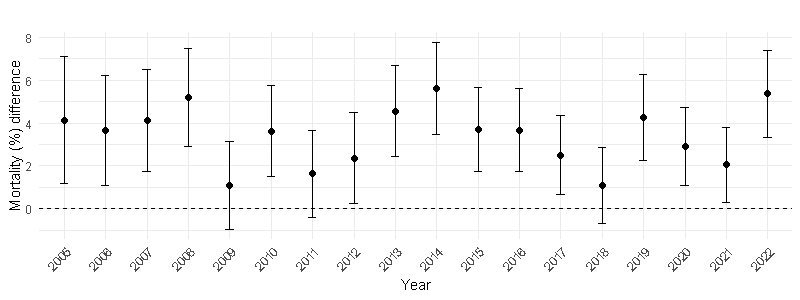


**eFigure 7.** Absolute in-hospital mortality (%) difference with 95 % confidence intervals between morning admissions and admissions during other times, categorised by admission year.


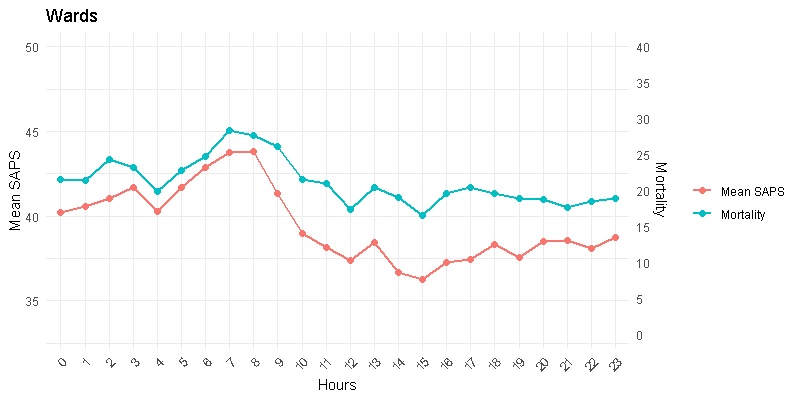


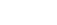


**eFigure 8.** Simplified Acute Physiology Score (SAPS) II and in-hospital mortality (%) according to admission hour for patients admitted from hospital wards. Adjusted odds ratio for in-hospital death for morning (6-12 a.m.) admissions, as compared to admissions during other times, 1.18 (95% confidence interval 1.10 – 1.27).


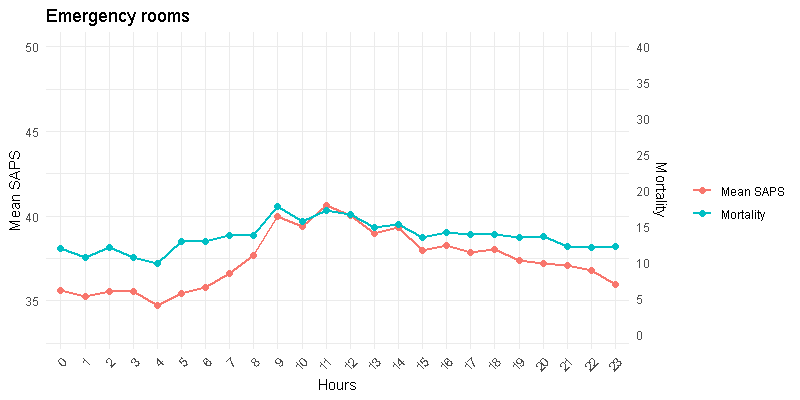


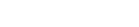


**eFigure 9.** Simplified Acute Physiology Score (SAPS) II and in-hospital mortality (%) according to admission hour for patients admitted from emergency departments. Adjusted odds ratio for in-hospital death for morning (6-12 a.m.) admissions, as compared to admissions during other times, 1.21 (95% confidence interval 1.14 – 1.28)


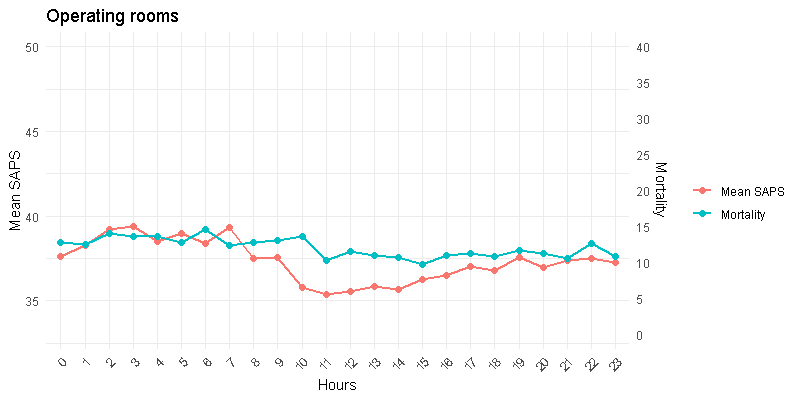


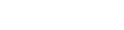


**eFigure 10**. Simplified Acute Physiology Score (SAPS) II and in-hospital mortality (%) according to admission hour for patients admitted from operating theatres. Adjusted odds ratio for in-hospital death for morning (6-12 a.m.) admissions, as compared to admissions during other times, 1.08 (95% confidence interval 0.98 – 1.18)


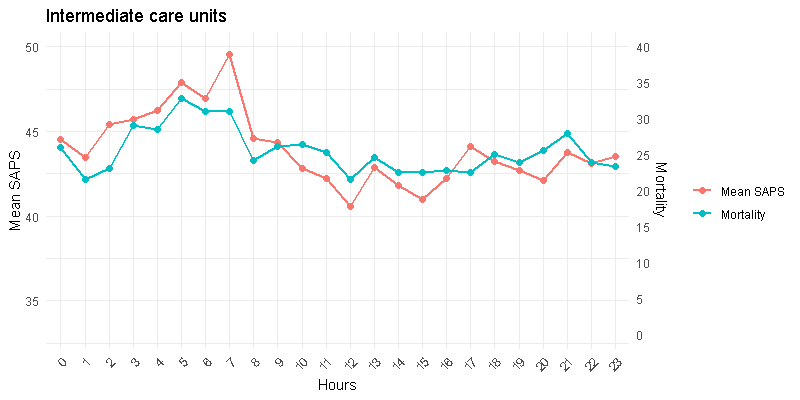


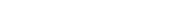


**eFigure 11.** Simplified Acute Physiology Score (SAPS) II and in-hospital mortality (%) according to admission hour for patients admitted from intermediate care units. Adjusted odds ratio for in-hospital death for morning (6-12 a.m.) admissions, as compared to admissions during other times, 1.06 (95% confidence interval 0.94 – 1.22)


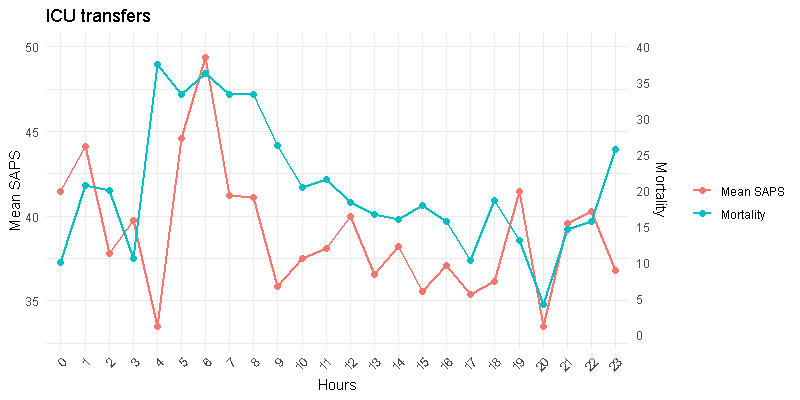


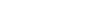


**eFigure 12**. Simplified Acute Physiology Score (SAPS) II and in-hospital mortality (%) according to admission hour for patients admitted after transfer from another intensive care unit. Adjusted odds ratio for in-hospital death for morning (6-12 a.m.) admissions, as compared to admissions during other times, 1.48 (95% confidence interval 1.06-2.05)

**eTable 1.** Amount of missing data among variables used in the study.

| Variable | Missing |
| --- | --- |
| Admission time | 0/131175 (0.0%) |
| Admission type (operative/non-operative) | 178/131175 (0.1%) |
| Age | 0/131175 (0.0%) |
| APACHE III diagnosis | 44/131175 (0.0%) |
| ICU category | 22/131175 (0.0%) |
| SAPS II score | 158/131175 (0.1%) |
| Sex | 0/131175 (0.0%) |
| Source of admission | 111/131175 (0.1%) |

_Data presented as absolute numbers and percentages (%); APACHE Acute Physiology and Chronic Health Evaluation; ICU, intensive care unit; SAPS II, Simplified Acute Physiology Score._

**eTable 2**. Adjusted odds ratios for in-hospital mortality for morning admissions (6-12 a.m.) and mean difference in Simplified Acute Physiology Score (SAPS II) between morning admissions and admissions during other times in different diagnostic categories.

| APACHE III category | OR (95% CI) | Mean difference (95% CI) |
| --- | --- | --- |
| Post-operative |  |  |
| Gastrointestinal | 1.09 (0.95 – 1.24) | -0.16 (-0.89 – 0.55) |
| Neurologic | 1.11 (0.90 – 1.35) | -0.61 (-1.63 – 0.41) |
| Respiratory | 1.21 (0.73 – 1.94) | 1.43 (0.09 – 2.77) |
| Trauma | 1.34 (0.97 – 1.83) | 0.20 (-1.12 – 1.52) |
| Vascular | 1.01 (0.83 – 1.21) | 0.85 (0.06– 1.70) |
| Other operative | 1.13 (0.58 – 2.07) | -0.59 (-1.79 – 0.59) |
| Medical (non-operative) |  |  |
| Cardiovascular/vascular | 1.09 (1.02 – 1.18) | 1.22 (0.57 – 1.89) |
| Gastrointestinal | 1.16 (1.03 – 1.31) | 1.87 (1.00 – 2.74) |
| Intoxication | 1.32 (0.82 – 2.08) | 0.81 (-0.16 – 1.79) |
| Metabolic/renal/hematologic | 1.26 (1.04 – 1.51) | 0.54 (-0.31 – 1.39) |
| Neurologic | 1.13 (1.01 – 1.27) | 1.03 (0.37– 1.69) |
| Respiratory | 1.26 (1.15 – 1.38) | 1.41 (0.89– 1.93) |
| Sepsis | 1.25 (1.11 – 1.42) | 1.25 (0.31 – 2.19) |
| Trauma | 1.45 (1.16 – 1.79) | -0.31 (-1.12 – 0.49) |
| Other non-operative | 1.47 (1.18 – 1.83) | 2.27 (1.19 – 3.35) |

_The regression model used to calculate mean difference for SAPS II score at admission included sex, source of admission (type of department where the patient came from), and diagnosis category as covariates. The model for in-hospital mortality included the same covariates and also age and admission type (post-operative or medical). Data presented as odds ratios (OR) and mean difference with 95% confidence intervals (CI). Classification based on_ _Acute Physiology and Chronic Health Evaluation (APACHE) III diagnostic categories._

**eTable 3.** Severity of illness adjusted odds ratios for in-hospital mortality between morning admissions (6-12 a.m.) and admissions during other times in different diagnostic categories.

| APACHE III category | OR (95% CI) |
| --- | --- |
| Post-operative |  |
| Gastrointestinal | 1.10 (0.95-1.28) |
| Neurologic | 1.13 (0.90-1.40) |
| Respiratory | 1.04 (0.58-1.80) |
| Trauma | 1.25 (0.87-1.78) |
| Vascular | 0.93 (0.75-1.15) |
| Other operative | 0.83 (0.39-1.66) |
| Medical (non-operative) |  |
| Cardiovascular/vascular | 1.02 (0.94-1.12) |
| Gastrointestinal | 1.04 (0.89-1.20) |
| Intoxication | 1.13 (0.67-1.83) |
| Metabolic/renal/hematologic | 1.11 (0.90-1.35) |
| Neurologic | 1.08 (0.94-1.23) |
| Respiratory | 1.21 (1.10-1.32) |
| Sepsis | 1.19 (1.03-1.37) |
| Trauma | 1.31 (1.01-1.69) |

_The regression model used to calculate odds ratios (OR) included SAPS II score, sex, source of admission (type of department where the patient came from), and diagnosis category as covariates. Data presented as ORs with 95% confidence intervals (CI). Classification based on Acute Physiology and Chronic Health Evaluation (APACHE) III diagnostic categories._

FINNICU research group

Matti Reinikainen 
Department of Anaesthesiology and Intensive Care, Kuopio University Hospital, and Institute of Clinical Medicine, University of Eastern Finland, Kuopio, Finland

Johanna Hästbacka  
Department of Intensive Care, Tampere University Hospital, Wellbeing Services County of Pirkanmaa, and Tampere University, Faculty of Medicine and Health Technology, Tampere, Finland

Anssi Pölkki 
Department of Anaesthesiology and Intensive Care, Kuopio University Hospital, and Institute of Clinical Medicine, University of Eastern Finland, Kuopio, Finland

Ville Ihalainen  
Department of Anaesthesiology and Intensive Care, Kuopio University Hospital, and Institute of Clinical Medicine, University of Eastern Finland, Kuopio, Finland

Salla Kattainen

Division of Intensive Care, Department of Perioperative and Intensive Care Medicine, University of Helsinki and HUS Helsinki University Hospital, Helsinki, Finland

Jenna Ehn

Department of Perioperative and Intensive Care, University of Helsinki and Helsinki University Hospital, Helsinki, Finland

Heikki Miettinen 
Department of Anaesthesiology and Intensive Care, Kuopio University Hospital, and Institute of Clinical Medicine, University of Eastern Finland, Kuopio, Finland

Pauliina Paananen 
Department of Anaesthesiology and Intensive Care, Kuopio University Hospital, and Institute of Clinical Medicine, University of Eastern Finland, Kuopio, Finland, and Turku University Hospital, Wellbeing Services County of Southwest Finland

Laura Mikkola 
Department of Anaesthesiology and Intensive Care, Kuopio University Hospital, Kuopio, Finland

Stepani Bendel 
Department of Anaesthesiology and Intensive Care, Kuopio University Hospital, and Institute of Clinical Medicine, University of Eastern Finland, Kuopio, Finland

Juuso Tamminen 
Wellbeing services county of South Karelia, Department of Anaesthesiology and Intensive Care, Kuopio University Hospital, and Institute of Clinical Medicine, University of Eastern Finland, Kuopio, Finland

Reijo Sund 
Institute of Clinical Medicine, University of Eastern Finland, Kuopio, Finland  
Clinical Research Center, Kuopio University Hospital, Kuopio, Finland

Tuomas Selander 
Science Service Center, Kuopio University Hospital, Kuopio, Finland

Heidi Urnberg 
Finnish Institute for Health and Welfare, Helsinki, Finland

Heikki Kiiski

Department of Intensive Care, Tampere University Hospital, Wellbeing Services County of Pirkanmaa, and Tampere University, Faculty of Medicine and Health Technology, Tampere, Finland
